# The Impact of Kohlrabi Sprouts on Various Thyroid Parameters in Iodine Deficiency- and Sulfadimethoxine-Induced Hypothyroid Rats

**DOI:** 10.3390/nu14142802

**Published:** 2022-07-08

**Authors:** Paweł Paśko, Krzysztof Okoń, Ewelina Prochownik, Mirosław Krośniak, Renata Francik, Jadwiga Kryczyk-Kozioł, Marta Grudzińska, Małgorzata Tyszka-Czochara, Mateusz Malinowski, Jakub Sikora, Agnieszka Galanty, Paweł Zagrodzki

**Affiliations:** 1 Department of Food Chemistry and Nutrition, Jagiellonian University Medical College, Medyczna 9, 30-688 Kraków, Poland; ewelina.gajdzik@uj.edu.pl (E.P.); miroslaw.krosniak@uj.edu.pl (M.K.); jadwiga.kryczyk@uj.edu.pl (J.K.-K.); malgorzata.tyszka-czochara@uj.edu.pl (M.T.-C.); pawel.zagrodzki@uj.edu.pl (P.Z.); 2 Department of Pathomorphology, Jagiellonian University Medical College, Grzegórzecka 16, 31-531 Kraków, Poland; k.okon@uj.edu.pl; 3 Department of Bioorganic Chemistry, Medical College, Jagiellonian University, Medyczna 9, 30-688 Kraków, Poland; renata.francik@uj.edu.pl; 4 Institute of Health, State Higher Vocational School, Staszica 1, 33-300 Nowy Sącz, Poland; 5 Department of Pharmacognosy, Jagiellonian University Medical College, Medyczna 9, 30-688 Kraków, Poland; grrudzinska@gmail.com (M.G.); agnieszka.galanty@uj.edu.pl (A.G.); 6 Department of Bioprocesses Engineering, Energetics and Automatization, Faculty of Production and Power Engineering, University of Agriculture in Krakow, Balicka 116b, 30-149 Kraków, Poland; mateusz.malinowski@urk.edu.pl (M.M.); jakub.sikora@urk.edu.pl (J.S.)

**Keywords:** kohlrabi sprouts, thyroid, histopathology, thyroid hormone, TSH, iodine deficiency, hypothyroidism, antioxidant capacity, cytokines, haematology, biochemical parameters

## Abstract

Brassica sprouts, as the rich source of dietary glucosinolates, may have a negative effect on thyroid function. In this study, kohlrabi sprouts diet, combined with two models of rat hypothyroidism, was tested. TSH, thyroid hormones and histopathology analysis were completed with the evaluation of immunological, biochemical, haematological parameters, cytosolic glutathione peroxidase, thioredoxin reductase in the thyroid, and plasma glutathione peroxidase. A thermographic analysis was also adapted to confirm thyroid dysfunction. The levels of TSH, fT3 and fT4, antioxidant enzyme (GPX) as well as histopathology parameters remained unchanged following kohlrabi sprouts ingestion, only TR activity significantly increased in response to the sprouts. In hypothyroid animals, sprouts diet did not prevent thyroid damage. In comparison with the rats with iodine deficiency, kohlrabi sprouts diet decreased TNF-α level. Neither addition of the sprouts to the diet, nor sulfadimethoxine and iodine deficiency, caused negative changes in red blood cell parameters, glucose and uric acid concentrations, or kidney function. However, such a dietary intervention resulted in reduced WBC levels, and adversely interfered with liver function in rats, most likely due to a higher dietary intake of glucosinolates. Moreover, the possible impact of the breed of the rats on the evaluated parameters was indicated.

## 1. Introduction

Plants, which are a popular element of daily diet, may have a beneficial or harmful influence on thyroid function. Such effect is associated with the presence of the so-called goitrogens—the compounds of different chemical structures, which reveal a significant impact on thyroid function in many ways. The most important classes of goitrogens are cyanogenic glucosides, sulphur compounds, namely, glucosinolates and their derivates, as well as flavonoids from isoflavones and C-glycosyl flavones subclasses, presented mostly in the cassava and flax products, brassica vegetables, soy and legume crops, millet and by-products, respectively [1]. However, it must be emphasized that these potential goitrogens may also have beneficial impact on the thyroid glands, such as antiproliferative effect on thyroid cancer cells, or the influence on the antioxidant processes in the thyroid itself [2,3,4].

Glucosinolates and their derivatives (isothiocyanates and thiocyanates), the active compounds from different parts of brassica vegetables (roots, bulbs, florets, sprouts, and even seeds), may have two faces. From one side, their regular and adequate intake is strongly associated with the decreased risk of different cancers, such as stomach, large intestine, rectum, breast, lungs, and prostate [5]. On the other hand, the same substances may be responsible for a harmful effect on thyroid function [1]. According to their anti-thyroid potential, brassica vegetables are categorized into three classes. The first group, with the highest risk of thyroid disruption and high content of progoitrin, includes Siberian kales and collards. The second group, represented by Chinese cabbages, is characterized by moderate risk and average progoitrin level, while the third one is associated with only minor goitrogenic effect as well as the lowest level of progoitrin (e.g., turnip, some Chinese and Japanese greens, broccoli). Interestingly, Brussels sprouts, according to different varieties, may be classified in the first or in the second group [6]. 

Kohlrabi (*Brassica oleracea convar. acephala* var. *gongylodes* L.) represents brassica vegetables, of which the bulbs are well known and often eaten, but the sprouts are a new example of functional foods. In our previous study, we evaluated the quality and quantity of glucosinolates in these products and we found that glucoerucin and glucoiberin were predominant compounds both in the bulbs and the sprouts, while sinigrin was detected only in the seeds. We also noted that sprouting time decreased the concentration of progoitrin in the sprouts harvested in natural light conditions, which varied from 0.06 ± 0.01 to 0.09 ± 0.02 mg/100 g fw [7]. This prompted us to focus on kohlrabi sprouts and expand our studies with the in vivo model. 

It should be strongly emphasized that most of the studies, which suggested negative effect of brassica vegetables, were conducted on animals (livestock and rodents) fed with brassica seeds, but not the edible parts of these vegetables (roots, leaves or bulbs), traditionally used in the daily diet. Until now, there are only few evaluations on the effect of brassica vegetables on thyroid function in human. A 12-week randomized clinical trial with broccoli sprouts beverage, administered to healthy volunteers [8], and a study with kale juice, administered to healthy adults [9], did not confirm the anti-thyroid effect of these products. On the contrary, a single case report described the development of myxoedema coma in a Chinese woman (88 years old), who consumed abnormal amounts of 1.0–1.5 kg of raw bok choy daily for several months [10]. 

The mechanism of the anti-thyroid effect of brassica vegetables is associated with two main crucial processes in the thyroid. Glucosinolates may inhibit the key proteins, such as sodium/iodide symporter (NIS), located in the basolateral membrane of thyroid follicle, responsible for iodide uptake, as well as the thyroid peroxidase (TPO), located on its apical membrane, which oxidizes iodide and controls the synthesis of thyroid hormones [1,9]. 

Because of these scarce and ambiguous results, mentioned above, the reliable evaluation of possible effect of regularly eaten brassica vegetables on the thyroid function is highly needed. Thus, based on our previous, promising results, concerning kohlrabi [7], we decided to evaluate the effect of kohlrabi sprouts in the in vivo animal model of hypothyroidism. The effect of the sprouts on particular immunological, biochemical, and haematological parameters of rats with developed hypothyroidism was assessed. Three dietary models were implemented in this study: (1) the standard diet, (2) the anti-thyroid diet with iodine deficiency (ID) causing thyroid hyperplasia [11,12], and (3) the anti-thyroid diet with sulfadimethoxine (SDM), added as an ingredient (0.025%) to the animal drinking water and causing thyroid damage by inhibiting thyroid hormone synthesis [13]. A broad spectrum of the parameters, describing the impact of the established treatments on rat organisms, was measured: (i) thyroid response parameters: serum concentration of thyroid-stimulating hormone (TSH), free thyroid hormones (triiodothyronine (fT3), and thyroxine (fT4)); (ii) haematological parameters: red blood cell count (RBC), haemoglobin (Hb), haematocrit (Hct), mean cell volume (MCV), mean cell haemoglobin (MCH), mean corpuscular haemoglobin concentration (MCHC), white blood cell count (WBC), thrombocyte count (PLT); (iii) biochemical parameters: glucose (Glu), uric acid (UA), urea (U), aspartate transaminase (ASPAT), alanine transaminase (ALAT), creatinine (Crea), triglycerides (TG), total cholesterol (TC), high-density lipoprotein (HDL), alkaline phosphatase (PAL); (iv) immunological parameters: interleukin 6 (IL-6), interleukin 10 (IL-10) and tumour necrosis factor alpha (TNF-α). Moreover, enzyme activities of cytosolic glutathione peroxidase (GPX1) and thioredoxin reductase (TR) in the thyroid tissue, ferric reducing ability of plasma (FRAP), plasma glutathione peroxidase (GPX3) activity were determined. The evaluation was completed with the measurement of rat body temperature.

## 2. Materials and Methods

### 2.1. Plant Material 

Kohlrabi seeds (*Brassica oleracea convar. acephala* var. *gongylodes* L.), Delikatess weisser cultivar, were collected from the plants harvested in north-west Poland in 2012. Voucher specimen was deposited in the Legutko Seed Testing Laboratory and in the Department of Food Chemistry and Nutrition, Faculty of Pharmacy, Jagiellonian University Medical College (No#BOAG/PP/PL 1043). Kohlrabi sprouts were provided by the Uniflora Company, Częstochowa, Poland and lyophilized to obtain dry material suitable for preparation of animal fodder. 

### 2.2. Animal Study 

The 4-week-old Wistar rats (72 male, mean weight 262.5 ± 8.2 g) were kept in plastic cages in an air-conditioned animal room in the Animal House of the Faculty of Pharmacy JUCM for one week before the experiment (temperature: 22 ± 2 °C; humidity of 50 ± 5%; 12 h periods of light and darkness). After 1 week of acclimatization, the rats were divided into 6 groups, each consisting of 12 animals, and fed with one of the following diets: a standard (control) diet (C); an iodine deficiency diet (ID); a diet with 7% of lyophilized kohlrabi sprouts (KS); an iodine deficiency diet with 7% of lyophilized kohlrabi sprouts (KS/ID); a standard diet with 0.025% SDM administered to animals with drinking water (SDM); or a diet containing 7% of lyophilized kohlrabi sprouts and 0.025% SDM administered in the drinking water (KS/SDM). The rats had unlimited access to fodder and water. The diets were prepared by The Morawski Fodder Company (Poland). Detailed descriptions of the diets composition were published previously [14], there were no significant differences in the mean volume of the drinking water between all evaluated groups, in case of the fodder intake the results were presented in Table 1. Water and fodder intake were monitored three times per week.

The protocols for animal experiments were approved by the Animal Experimentation Committee of Jagiellonian University, Kraków, Poland (No. 76/2014). After 8 weeks, the blood was collected from the abdominal aorta under thiopental anaesthesia (60 mg/kg bw ip.) for hormone assays and other parameters determinations, apart from haematological parameters, which were evaluated as described further. Prior to the analyses, the samples were stored at −80 °C. Thyroid glands were collected and divided to be analysed for the following parameters: GPX1 and TR activities and for histopathological examination.

### 2.3. TSH, fT3, fT4 Analysis

Thyroid hormone analyses of serum free T4 (fT4), free T3 (fT3) and TSH levels were performed with immunoassay kits (DRG MedTek, Warsaw, Poland), according to the manufacturer’s instructions. The methods have been validated for rat serum. An automatic reader (Synergy-2, BioTek/Winooski, VT, USA) was used in the immunoassays. Hormone analyses were evaluated for all rats (*n* = 12) in all groups. The concentrations of fT4, fT3 and TSH were presented as ng/dL, pg/mL and μIU/L, respectively.

### 2.4. Thyroid Histopathology Analysis

The specimens were fixed in 10% buffered formalin, sliced with a microtome blade into 2 mm thick sections, processed by routine histologic method and embedded in paraffin. From the paraffin blocks, 2 µm single sections were prepared and stained by routine haematoxylin and eosin method. The assessment of histology was completed in a blinded manner, i.e., without the knowledge of the study groups. Both qualitative description and image acquisition were performed with Axioscop microscope (Zeiss GmbH, Oberkochen, Germany). The measurements were made as previously described [15]. All the sections containing recognizable thyroid tissue were photographed using PlanNeoFluar 2.5× lens and Nikon D5300 camera. For calibrating the images, a 1 mm microscopic grid (produced by Zeiss Jena, Germany) was photographed with the same microscopy system. The images were transferred to standard personal computer and processed interactively using Photoshop CS4 (Adobe Systems Inc., San Jose, CA, USA) software to leave only thyroid follicles, and marking the background, non-thyroid tissues as well as thyroid colloid as white. The resulting images were saved as files under altered filenames. Measurements were made with AnalySIS 3.2 pro (Soft Imaging Systems GmbH, Münster, Germany) image analysis system controlled by a program written by one of the authors (K.O.) in Imaging C macro language. The program loaded the successive image files, recorded the surface area of the thyroid tissue as well as area of the empty ‘holes’ which constituted the lumens of thyroid follicles. Before processing the batch of images, the system was calibrated using the reference images. The results were saved to a text file.

### 2.5. Enzyme Activity and Antioxidant Plasma Capacity Analysis

The methods for determining parameters such as FRAP, GPX, TR were described in our previous papers [4,15] and appropriately adapted to 48-well or 96-well plates [16]. The thyroid tissue samples were homogenized in phosphate buffer pH = 7.4. The GPX3 and FRAP were evaluated in the plasma. The change of absorbance during FRAP determination was measured after 8 min. of incubation and the reducing ability of the sample was expressed as μmol Fe^2+/^L of plasma. The TR and GPX1 were investigated in the thyroid tissue. Protein content was determined by Bradford method (BioRad, Hercules, CA, USA). In all of the above-mentioned methods, an automatic reader Synergy-2, BioTek, Winooski, VT, USA was used. All the parameters indicated above were determined for all the rats (*n* = 12) in each group. For GPX1 and TR investigation in thyroid glands, analyses were conducted in 4 rats in each group, due to the small size of the thyroid material. The activities of the evaluated enzymes were presented as U/g, U/mg, and mU/mg of protein for GPX1, GPX3 and TR, respectively.

### 2.6. Measurement of Cytokine Levels

Rat IL-6, IL-10 and TNF-α ELISA kits were obtained from Diaclone (Besançon, France) and the determination of the levels of these cytokines were performed according to the manufacturer’s instructions using an automatic reader Synergy-2, BioTek/USA. Cytokine determinations were performed for 6 rats per group and expressed as pg/mL.

### 2.7. Biochemical Analysis

Plasma biochemical analyses were performed with appropriate commercial kits (Biomerieux, Marcy-l’Étoile, France), and in accordance with the manufacturer’s instructions. An ALIZE automatic biochemical analyser (Lisabio, Pouilly-en-Auxois, France) was used. Biochemical parameters were evaluated for each rat (*n* = 12) in all the groups. The concentration of Glu, TG, TC, HDL, U was presented as mmol/L, and μmol/L in the case of creatinine. ASPAT, ALAT and PAL activity were expressed as U/L, and UA concentration as mg/dL.

### 2.8. Haematological Evaluation

Blood samples of about 600 μL were obtained from rat tail veins and placed in plastic Microvette 100 K3E tubes (Sarstedt, Nümbrecht, Germany). A complete blood count was performed using an ABX COBAS MICROS Haematology automated cell counter (ROCHE, Basel, Switzerland). The following parameters were determined in all animals (*n* = 12) in each group: RBC, Hb, Hct, MCV, MCH, MCHC, WBC and PLT, presented then as 10^6^/µL, g/dL, %, fL, pg/cell, g/dL, 10^3^/µL and 10^3^/µL, respectively.

### 2.9. Thermographic Investigation 

The body temperature was registered at the end of experiment. A thermographic camera (ThermaCAM e300) manufactured by FLIR (Täby, Sweden), with a thermographic resolution of 0.1 °C was used. Thermographic images were taken from 1 m distance in the long wave range (7.5–14 μm). Each pixel in the thermogram represents an area of 1.6 mm^2^. The results of thermographic measurements are presented as digital pictures (thermograms) which constitute a database where different temperature values are represented with different hues. Such an analysis was conducted between 8 a.m. and 11 a.m. under similar lighting, temperature and relative humidity conditions. Thermograms were composed with QuickReport 1.2 and Reporter 2000 Pro software (FLIR System AB, Täby, Sweden). The influence of the ambient temperature and other parameters was taken into account while scrutinizing the data.

### 2.10. Statistical Analysis and Chemometric Approach

All quantitative data are shown as mean values ± standard deviations. Comparisons between groups were performed using Kruskal–Wallis test, with Dunn Post Hoc Test.

A partial least square model (PLS) was applied to describe the correlation structure between parameters in a whole group of animals. The details of our approach to PLS model were described in our previous papers [4,17,18]. Statistical analyses were performed using the packages: Graph Pad Prism v.3.02 (GraphPad Software, San Diego, CA, USA), STATISTICA v. 13.3. (TIBCO Software Inc., Palo Alto, CA, USA) and SIMCA-P v.9 (Umetrics, Umeå, Sweden). The correlation weights were calculated using software delivered by MP System Co. (Chrzanów, Poland).

## 3. Results and Discussion

### 3.1. Hormones Level and Histopathology Analysis

The influence of kohlrabi sprouts on thyroid function in vivo, confirmed by the selected parameters associated with hormone synthesis, enzyme activity, crucial for this organ function, histopathology, and TSH, was evaluated for the first time. In our study, we implemented two different animal models of hypothyroidism (deficiency of iodine and inhibition of TPO activity by sulfadimethoxine). All these results are gathered in Table 2 and Table 3.

In the animals with model thyroid damage, significant increase in TSH levels was noted in the sulfadimethoxine model, in comparison with the control group, while in case of iodine deficiency model, only increasing tendency of this parameter was observed. For fT3 level, a significant decrease (*p* < 0.01) was found in SDM diet group, but in case of fT4, there were no changes in both implemented models. Regarding the thyroid damage model itself, two important remarks were noted, when we compared the present results with our previous observations, referring not only to the time of the experiment, but also to rat breed used. As far as the time of hypothyroidism induction is concerned, 5 weeks of experiment caused significant increase in TSH level and the decrease in T4 level, with no effect in T3, but for 10 weeks of experiment, no differences in TSH and T3 levels, and only significant decrease in T4 was noted in the iodine deficiency model [11,13]. The compensatory increase of TSH is recognized as the earliest indicator of hypothyroidism [19], according to the negative feedback between TSH and thyroid hormones. It was previously suggested that in case of the iodine deficiency, lack of changes in TSH levels may result from the effect of the adaptation of the animals, observed in the experiments lasting 8 weeks or longer [4,11]. It is interesting that 8 weeks of treatment had no influence on the fT4 concentration, as it was also confirmed for older pigs, treated with goitrogens and with iodine depletion [20]. The T4 level was only decreased in young, 4-week-old pigs. Sulfadimethoxine as an anti-thyroid compound seems to be a stronger agent than the deficiency of iodine, causing the inhibition of thyroid peroxidase and thus a significant decrease of fT3 and the increase of TSH [4,13]. We also observed that the rats of different breeds may reveal varied response to the thyroid disrupting agents and the summarizing remarks and comments on this phenomenon are presented in Section 2.2. The applied models were approved and confirmed for the evaluation of food impact on thyroid function [11,12,13]. 

The inclusion of kohlrabi sprouts into the diet of healthy rats did not have any significant influence on the level of TSH, fT4, and fT3, which indicates the high safety profile of these sprouts in the context of normal thyroid function. Moreover, no enhancement in the negative effect on thyroid hormones and TSH level was observed during the intake of kohlrabi sprouts by rats with iodine deficiency or sulfadimethoxine ingestion. It should be emphasized that the lack of the negative effects of kohlrabi sprouts is probably associated with low content of progoitrin, the most potent goitrogen in brassica vegetables. The observed safety profile of kohlrabi sprouts agrees with the classification of brassica vegetables, according to their goitrogenic properties, proposed by Langer et al. [6]. It should also be highlighted that our results bring some new arguments, supporting the hypothesis that brassica vegetables are not so dangerous, in terms of their impact on the thyroid, as it was previously described in some outdated studies, based on the effect of seeds which are not typical element of human diet [20,21,22,23]. However, contrary to our expectation, no protective effects of kohlrabi sprouts on thyroid damage was found, while such an effect was observed in our previous study for rutabaga sprouts, with significant decrease of TSH and no effect on fT3 and fT4 levels in rats with iodine deficiency [15], or for broccoli sprouts, with a protective effect in sulfadimethoxine-damaged thyroid [4].

**Figure 1:**
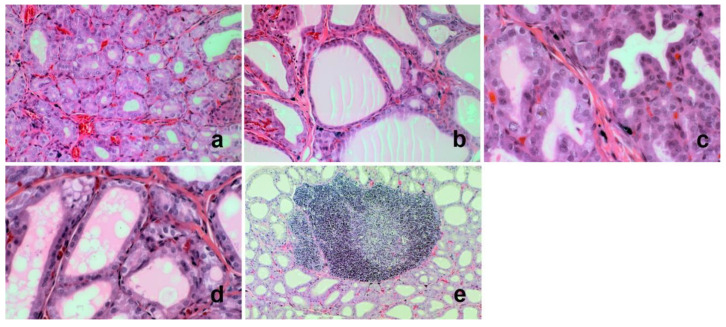
Histopathology of selected rat thyroid glands: (**a**) Cuboidal follicular epithelial cells lining small follicles. Haematoxylin and Eosin (H + E) original magnification 200×. (**b**) Flattened follicular epithelial cells lining larger follicles. H + E original magnification 200×. (**c**) Few small papillary projections of follicular epithelial cells protruding into the lumina of the follicles. H + E original magnification 400×. (**d**) Vacuolation of the colloid. H + E original magnification 400×. (**e**) Lymphoid follicles. H + E original magnification 400×.

The effect of kohlrabi sprouts on thyroid gland histopathology has been described for the first time. During histopathology analysis of thyroid glands, papillary formation, aggregates of lymphocytes, vacuolization of the colloid, shape of epithelial cells, follicular epithelial area, follicular luminal area, and overall thyroid area were evaluated (Table 3 and Figure 1). The highest significant overall thyroid area was noted in the SDM group. Additionally, the highest (*p* < 0.05) follicular epithelial area was observed in the SDM and KS/SDM group. Higher papillary formation appeared in the SDM group and increased concomitantly in the KS/SDM group. Low papillary formation was noted frequently in KS/ID animals, in comparison with control group and KS group. Only in the ID diet group, the vacuolization process in thyroid cells was observed as also the highest follicular luminal area in comparison with control group (*p* < 0.05) was noted. Specifically, in the control group thyroid follicles were lined by flattened epithelial cells. In the ID and SDM groups, the follicles were lined by cuboidal cells in about 60% and 100% of evaluated thyroids from each group, respectively. The morphological changes observed in SDM and ID groups were correlated with significant increase or increasing tendency of TSH, respectively, which is one of the most important factors controlling the morphofunctional status of each follicle [24]. For the KS/SDM or KS/ID groups, the follicles were lined by cuboidal cells in all animals from the KS/SDM group and in 50% of animals from KS/ID group. The correlation weights for the pairs of parameters based on PLS model, including histopathological parameters were calculated. The negative correlation weights were observed between fT3 and follicular epithelial area (−0.147) and overall thyroid area (−0.148).

Kohlrabi sprouts, included in the diet, increased follicles extensive hypertrophy only in SDM group, but no presence of papillae was observed. Additionally, the aggregates of lymphocytes were detected only in KS group (25% of animals). In contrast, rutabaga sprouts caused lining follicles by cuboidal cells in only about 40% of evaluated thyroids [15]. Additionally, the opposite effect was observed in case of follicles extensive hypertrophy, where rutabaga sprouts cause the same effect in ID group and the animals receiving the sprouts and SDM, while in healthy rats, papillae were present in some cases [15]. Some papillary formation for field mustard diets was noted previously [25], with the highest development in rats fed with rape seeds. For rutabaga sprouts, lymphocyte aggregates were observed, but without any significant changes in their levels and they coincided with high papillary formation only in the ID diet animals fed with rutabaga sprouts [15]. 

### 3.2. Antioxidant Effect

Thyroid hormone synthesis requires hydrogen peroxide for iodine organification. Because of this process, thyroid also needs some mechanisms, such as the activity of GPX, CAT and thioredoxin reductase, to be protected from the oxidation [26]. Thus, in the next step of the experiment, we wanted to verify whether the experimental treatment can influence the activity of those protecting enzymes. In this experiment, there were no changes in GPX1 and GPX3 activity, but significant increase in case of TR activity was noted in the KS group, when compared to the control animals. Antioxidant activity of plasma, measured by the FRAP method, was significantly higher in the KS/SDM group, in comparison with C, ID and KS/ID groups. Similar effects were previously found for broccoli and rutabaga sprouts, it was even suggested that the increasing activity of this enzyme may be one of the beneficial effects of sulphur compounds in brassica products [4,15,27]. 

### 3.3. Immunological Parameters 

Thyroid damage induced by iodine deficiency may subsequently initiate proinflammatory response of the gland. Such an effect was observed in the next step of our experiment, manifested as the significant increase in IL-6 in ID vs. C group and increasing tendency in TNF-α. As far as the SDM group is concerned, only slight increasing tendency in both parameters was noted. In case of proinflammatory cytokines production, some adaptive changes may be observed, especially in the mild course of inflammation during hypothyroidism. It is interesting that the addition of kohlrabi sprouts partially limited the inflammation processes, induced by iodine deficiency, significantly reducing TNF-α in the KS/ID vs. ID groups. For IL-6, only a slight positive effect in this aspect was noted. Unexpected results were noted in case of anti-inflammatory IL-10 level, which decreased significantly in the KS/SDM vs. SDM groups. It should not be excluded that kohlrabi sprouts may modulate the inflammation process through other paths. Our observation is in agreement with Abbas et al. [28], who found a significant increase in IL-6 and TNF-α in the animals with methimazole-induced hypothyroidism. Lack of significant influence of hypothyroid state on TNF-α level in our study can be explained by the longer period of the study (8 weeks), in contrast with the methimazole model, which lasted 4 weeks. Similarly, in our previous study, we noted that broccoli sprouts caused only a slight decrease in this cytokine level when administered to rats with iodine deficiency [4]. However, in case of rats receiving rutabaga sprouts, an increasing tendency in the concentration of IL-10 was observed for the ID and SDM animals [17]. The observed anti-inflammatory potential of kohlrabi may result from the presence of different active compounds. Lee et al. [29] observed the decrease in nitric oxide production in lipopolysaccharide-induced RAW 264.7 murine macrophages pre-treated with 3-(3,4,5-trimethoxyphenyl)-2E-propenoic acid methyl ester; (E)-sinapoyl glucoside, and lawsoniaside B, isolated from red kohlrabi sprouts. Red bulbs of kohlrabi presnted anti-inflammatory activity, confirmed by the downregulation of the iNOS and COX-2 production in the in vitro model [30]. Additionally, we observed that the strong negative correlation between IL-6 and fT3 was revealed by the PLS model (CW = −0.141), which supports the hypothesis of Contreras-Jurado et al. [31] about inhibiting influence of T3 on the cytokine production and is in agreement with Davies et al. [32] and Quispe et al. [33].

### 3.4. Biochemical Parameters 

In biochemical analysis of hypothyroid animal models, ten parameters were evaluated (see Section 2.7 for details). Significant differences were noted only in case of creatinine, ALAT, ASPAT, TG, TC and PAL (Table 4). In the ID diet group, significant increase of ALAT and PAL was observed, while in the SDM diet animals, these parameters were not significantly changed, but a decrease in ASPAT was noted, when compared with the control animals (C vs. SDM *p* < 0.01). The KS diet, in comparison to the control group, increased significantly ALAT and PAL, while the decrease in ASPAT was not significant. It is also interesting that the PLS model applied in our study revealed a positive correlation between ASPAT and fT3. Our results are in the opposition to Messarah et al. [34], who did not find any changes in these parameters in rats with hypothyroidism. However, in our previous studies, broccoli sprouts caused similar changes in biochemical parameters of liver function [4], but rutabaga sprouts decreased ASPAT and ALAT without the influence on PAL [17]. It was suggested that high consumption of brassica vegetables may increase significantly the activity of different isoforms of CYP because of the glucosinolates content [35]. Additionally, Hasan et al. [36] observed a significant increase in ASPAT, and PAL in rats fed with rapeseeds fodder, but the effect on ALAT was dependent on the origin of the seeds (wild or hybrid). However, Abouzed et al. [37] noted a significant decrease in ALAT in rats with hepatocellular carcinoma after the ingestion of S-methyl methionine sulfonium chloride (50 mg/kg/day)—a methionine derivative presented in brassica vegetables.

It Is known that thyroid hormones may influence the total cholesterol, LDL, HDL, ApoB and triglycerides levels. This effect is partially associated with the activity of HMG-CoA reductase; sterol response element binding protein, CYP7a1, acetyl-CoA carboxylase, fatty acid synthase and carnitine palmitoyl transferase-Iα [38]. Increase in TG in subclinical hypothyroidism is not always observed [39]. In our experiment with kohlrabi sprouts, we observed a significant decrease of TG and TC in the ID diet group, in comparison to C, KS, KS/ID and C, KS/ID, SDM, KS/SDM, respectively, but the explanation for this phenomenon requires further study. Nevertheless, kohlrabi sprouts seem to not be a significant element of the diet, in terms of the influence on the lipid profile, which was also confirmed in the experiment by Gao et al. [40], where feeding of steers with rapeseed cakes, containing high amount of glucosinolates, did not have any significant effects on the TC and TG profile. Our previous study with Fischer (F344/DuCrI) rats showed significant increase in TG level, but not for TC in hypothyroidism. This strongly supports our idea that the breed of rats is very important in these types of the experiments [17]. Glucose, uric acid, and urea were not changed in any of the groups in our experiment, only creatinine level decreased in hypothyroid rats (ID, KS/ID), significantly in the latter. No effect on glucose and urea was noted in our previous study [4,17]. Hasan et al. [36] observed a significant increase in creatinine after rapeseed ingestion in rats, but for the steers fed with rapeseed cakes, where only urea and glucose were evaluated, no changes were found [40].

### 3.5. Haematological Parameters

Sulfadimethoxine or its metabolites, such as hydroxylamine or nitroso derivatives, may cause leukopenia, associated with hypersensitivity reactions [41]. Thyroid gland removal in newborn or young adult rats caused a reduction in the number of peripheral blood lymphocytes [42], which indicates that properly functioning thyroid is crucial for the normal lymphocytes level in the organism. Consequently, both models of hypothyroidism caused significant decrease in WBC level, with simultaneously observed decreasing tendency in PLT amount. Similarly, kohlrabi sprouts diet, both alone or in combination with ID and SDM diet, caused a significant decrease in WBC and slight in PLT levels, in comparison to the control group. On the contrary, our results indicated that neither the addition of kohlrabi sprouts to the diet, nor sulfadimethoxine or iodine deficiency exposure caused significant changes in RBC and MCH parameters. 

In a similar study with canola seeds, broccoli, and rutabaga sprouts, no adverse effect on haemoglobin and haematocrit in broilers and rats was noted [4,17,43]. What is interesting, similar significant decrease in WBC and PLT was observed for rats fed with broccoli sprouts, while for rutabaga sprouts, only a decreasing tendency in WBC and PLT was observed [4,17]. It was also found that the consumption of 20 g broccoli sprouts for 4 days caused a significant reduction in the number of lymphocytes, and in the percentage of monocytes, in 23 patients with no history of chronic illnesses [44].

### 3.6. Body Temperature Determination 

The results for the changes in thyroid-related parameters, reported above, have been finally completed with the evaluation of body temperature of the examined animals (see Table 2). Neither kohlrabi sprouts diet, nor iodine deficiency diet, caused significant changes in the body temperature, when compared to the control conditions. Significant decrease in this parameter was observed in the following groups, in comparison to control rats: SDM (*p* < 0.001); KS/SDM (*p* < 0.01), and KS/ID (*p* < 0.05). The lowest body temperature was observed in SDM group (31.7 ± 1.8 °C), for which also fT3 level was the lowest, when compared to all the investigated animals. This observation was further confirmed by significant positive correlation between these two parameters (CW = 0.155), which is in accordance with the fact that T3 increases the metabolic rate by inducing transcription of uncoupling protein 1 and influencing the thermogenic program [45]. Kohlrabi sprouts administered to the ID diet animals led to a more profound temperature decrease, when compared to the control animals and the ID diet itself. This may suggest that sprouts intake enhances the effect of iodine deficiency diet, while the opposite pattern was observed in the SDM group, where a positive increasing trend in body temperature was found. Broccoli sprouts administered to hypothyroid rats revealed the same effect, but the differences were significant [4].

### 3.7. Chemometric Analysis 

**Figure 2:**
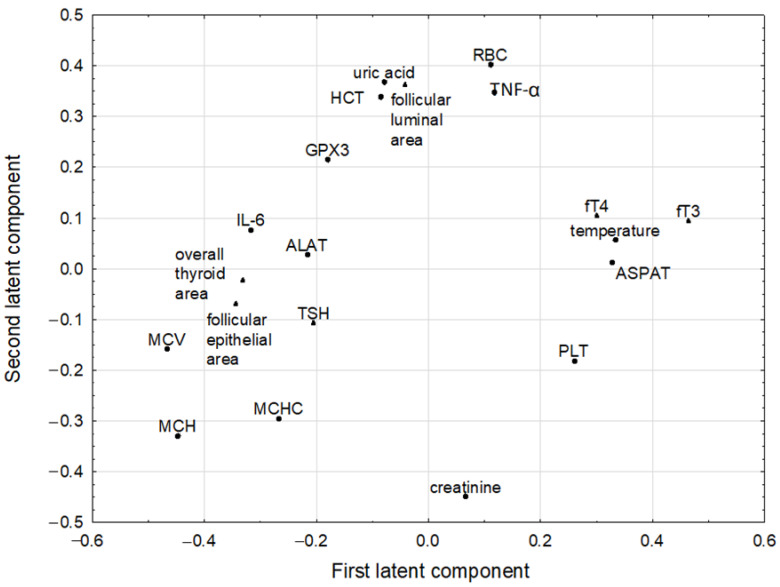
The loadings for first two latent component of PLS model (the predictive parameters are indicated by dots and the response parameters by triangles), see Section 2. for the explanation of all abbreviations.

The PLS model fulfilling cross-validation criteria was constructed. The predictive parameters were: RBC, HCT, MCV, MCH, MCHC, PLT (blood parameters), ASPAT, ALAT, creatinine, temperature (comprehensive metabolic panel parameters), uric acid, plasma GPX (parameters characterizing antioxidant status), IL-6, TNF-α (immunological parameters), while fT3, fT4, TSH, follicular epithelial area, follicular luminal area, overall thyroid area (parameters characterizing thyroid function) were selected as response parameters. Other parameters were not included in the model as they were considered noninformative. The model had four significant components, and explained 67.3% of variance in the predictive parameters, and 54.5% of variance in the response parameters, with eigenvalues of 3.54, 3.33, 1.39 and 1.17 for subsequent components, respectively. The loadings for first two latent components are shown in Figure 2. 

The first latent component in this model had positive weights predominantly for the fT3, temperature, ASPAT, and negative weights for MCV, MCH, follicular epithelial area, overall thyroid area and IL-6. The highest positive correlation weights based on this latent component were revealed between MCV and MCH, MCV and follicular epithelial area, temperature and fT3, ASPAT and fT3, MCV and overall thyroid area, while negative between MCV and fT3 (Table 5). The second latent component was loaded mainly by RBC, uric acid and follicular luminal area (positively) and by creatinine (negatively) (Figure 2). Therefore, RBC and uric acid had high correlation weight as well as RBC and follicular luminal area. All three parameters correlated negatively with creatinine. Among strong correlations, there were also negative correlation between MCV and fT3, between the latter parameter and follicular epithelial area, the overall thyroid area and IL-6 (Table 5).

### 3.8. The Importance of the Breed of the Rats Used in the Experiment 

The determination of the biological effects of three types of brassica sprouts (rutabaga, broccoli, kohlrabi), as the examples of functional foods with the special influence of on thyroid function, was the aim of our extensive project, led for the last several years [4,7,15,17,46,47,48], with kohlrabi sprouts study as the final step. To sum up this evaluation, we decided to collect the most important data about the influence of the breed of rats on the observed effects in two applied in vivo different models of hypothyroidism (Table 6). Additionally, in Table 7, we presented the differences in the response of the different breed of healthy animals on the sprouts’ ingestion alone. In the case of Wistar rats, SDM diet caused a significant increase in the level of TSH and the decrease of fT3 [4], while in Fischer (F344/DuCrI) rats, the same model influenced significantly only TSH level. The ID model diet was responsible for fT3 decreasing only in Fischer rats [15], without any effect in Wistar rats. Such an observation, made for the first time, may be of great importance when referring to the comparison of the results presented by different authors, and drawing the final conclusions from the experiments performed of rats differing in the breed.

Such final compilation of all the results about three types of brassica sprouts and their effects on the functioning of the organisms may be an interesting and helpful tool for designing future in vitro and in vivo studies in this area. This knowledge may be useful also for the evaluation of the safety profiling of functional foods.

## 4. Conclusions

**Figure 3:**
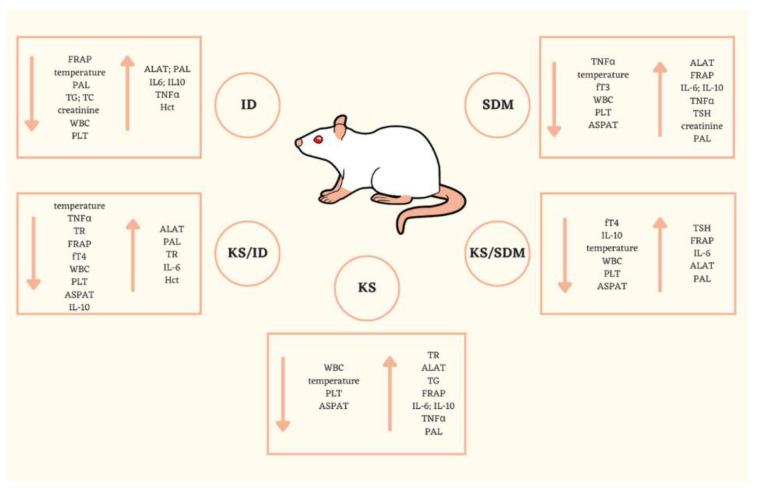
Summary of the main findings (significant changes and tendencies) describing the effect of hypothyroid models and kohlrabi sprouts diet on Wistar rats, see Section 2. for the explanation of the groups’ acronyms and all abbreviations; ↑increase; ↓decrease.

We have demonstrated for the first time that the kohlrabi sprouts diet did not cause any significant harmful effect on the thyroid function of healthy male rats. However, no protective effect of kohlrabi sprouts against thyroid damage was observed. What should be noted is that kohlrabi sprouts decreased the number of white blood cells and adversely interfered with liver function in healthy rats (Figure 3). Our results strongly suggest that neither the addition of kohlrabi sprouts to the diet, nor sulfadimethoxine or iodine deficiency exposure, caused negative changes in red blood cells parameters, glucose level, and uric acid. All these results may be useful in confirming the safety of kohlrabi sprouts. For the first time, the significance of the rats’ breed on the effectiveness and response of hypothyroidism model was noted.

It is important to underline that the obtained results are valid for young male rats’ thyroid function, and the translation of these results to potential human exposure to kohlrabi sprouts could result in some misinterpretation. Furthermore, until new data confirm a lack of any negative effect of brassica sprouts on thyroid function in humans, this product should not be excluded from the group of goitrogens, especially for people with coexisting iodine deficiency.

## Figures and Tables

**Table 1:** The mean fodder intake by the rats of different experimental groups (*n* = 12, g/day).

C	KS	ID	KS/ID	SDM	KS/SDM
14.8 ± 3.3 ^abc^	11.0 ± 2.8 ^a^	11.75 ± 3.0 ^de^	13.7 ± 2.9 ^f^	23.1 ± 3.8 ^bdefg^	12.3 ± 2.9 ^cg^

Mean values with the same superscript are significantly different between the indicated groups at *p* < 0.01 (a,c); *p* < 0.001 (b,d,e,f,g). See Section 2.2 for the explanation of the groups’ acronyms.

**Table 2:** Hormone levels, antioxidant status parameters of thyroid glands, plasma level of interleukins, and body temperature of the investigated rats (see Section 2. for the explanation of the groups’ acronyms and all abbreviations).

Parameters	C	KS	ID	KS/ID	SDM	KS/SDM	*p* Value
**Hormones**							
TSH [μIU/L] *n* = 12	9.15 ± 1.55 ^ab^	9.44 ± 1.51 ^c^	10.43 ± 2.71	9.26 ± 1.52 ^d^	29.86 ± 19.43 ^acd^	23.23 ± 13.37 ^b^	^a^ *** ^d^ ** ^bc^ *
fT3 [pg/mL] *n* = 12	4.33 ± 0.70 ^a^	4.25 ± 0.99 ^b^	4.52 ± 0.99 ^c^	4.24 ± 0.99 ^d^	2.91 ± 0.93 ^abcd^	3.19 ± 1.17	^ac^ ** ^bd^ *
fT4 [ng/dL] *n* = 12	10.78 ± 4.12	9.03 ± 1.84	10.65 ± 1.81 ^a^	8.34 ± 1.67	9.09 ± 2.81	8.06 ± 1.61 ^a^	^a^ *
**Thyroid Glands**							
GPX1 [U/g] *n* = 4	2.27 ± 0.99	3.58 ± 0.54	3.53 ± 1.24	2.62 ± 0.76	1.54 ± 0.40	2.39 ± 0.74	^-^
TR [mU/mg] *n* = 4	2.54 ± 0.13 ^a^	5.89 ± 3.20 ^a^	3.51 ± 2.32	4.35 ± 2.62	1.86 ± 0.87	2.46 ± 0.72	^a^ *
**Plasma**							
GPX3 [U/mg] *n* = 12	0.50 ± 0.17	0.53 ± 0.04	0.56 ± 0.03	0.50 ± 0.03	0.51 ± 0.04	0.51 ± 0.04	^-^
FRAP [μmol/L] *n* = 12	434.6 ± 88.9 ^a^	468.5 ± 66.6	391.7 ± 39.0 ^b^	430.6 ± 36.0 ^c^	475.7 ± 89.2	594.2 ± 148.1 ^abc^	^b^ *** ^ac^ **
IL-6 [pg/mL] *n* = 6	20.50± 10.74 ^a^	31.94 ± 10.93	54.73 ± 9.28 ^a^	26.77 ± 16.53	46.04 ± 24.96	43.83 ± 3.61	^a^ *
IL-10 [pg/mL] *n* = 6	32.06 ± 8.52	37.44 ± 7.13	60.76 ± 25.86	21.06 ± 4.13	78.46 ± 48.12 ^a^	16.32 ± 9.27 ^a^	^a^ *
TNFα [pg/mL] *n* = 6	45.80 ± 32.25	65.25 ± 29.10	71.67 ± 9.63 ^a^	13.40 ± 6.24 ^a^	53.72 ± 29.55	42.00 ± 17.62	^a^ *
**Body Temperature**							
TEMP [°C] *n* = 12	35.5 ± 0.6 ^abc^	33.2 ± 0.7	34.3 ± 1.7	32.7 ± 1.4 ^a^	31.7 ± 1.8 ^b^	32.7 ± 1.3 ^c^	^a^ * ^b^ *** ^c^ **

Mean values with the same superscript are significantly different between the indicated group at * *p* < 0.05; ** *p* < 0.01; *** *p* < 0.001.

**Table 3:** Qualitative and quantitative parameters describing the changes observed during histopathology of thyroid glands of the investigated rats (see Section 2.2. for the explanation of the groups’ acronyms).

Parameters	C	KS	ID	KS/ID	SDM	KS/SDM
**Papillary formation [%]**	0%	0%	0%	25%	60%	80%
**Aggregates of lymphocytes [%]**	0%	25%	0%	0%	0%	0%
**Vacuolization of the colloid [%]**	0%	0%	40%	0%	0%	0%
**Shape of epithelial cells [flatted vs. cuboidal] [%]**	100% flatted	60% cuboidal	60% cuboidal	50% cuboidal	100% cuboidal	100% cuboidal
**Follicular epithelial area [mm^2^]**	1.57 ± 0.14 ^ab^	1.77 ± 0.86 ^c^	3.06 ± 0.71	1.59 ± 0.82 ^d^	4.37 ± 1.03 ^bcd^	3.57 ± 1.68 ^a^
**Follicular luminal area [mm^2^]**	0.32 ± 0.06 ^a^	0.38 ± 0.14	0.69 ± 0.24 ^ab^	0.41 ± 0.20	0.41 ± 018	0.22 ± 0.06 ^b^
**Overall thyroid area [mm^2^]**	1.79 ± 0.25 ^a^	2.24 ± 0.80	3.50 ± 1.13	1.97 ± 1.00 ^b^	4.64 ± 1.40 ^ab^	3.79 ± 1.72

[%] of animals in which the changes were observed. Results marked with the same letters within each row differ significantly (*p* < 0.05).

**Table 4:** The blood morphology and biochemical parameters of rats (see Section 2. for the explanation of the groups’ acronyms and all abbreviations).

Parameters	C	KS	ID	KS/ID	SDM	KS/SDM	*p*-Value
*n* = 12	**Blood Morphology Parameters**						
RBC [10^6^/μL]	9.13 ± 0.60	9.21 ± 0.92	9.89 ± 0.45 ^a^	9.32 ± 0.81	9.13 ± 0.45 ^a^	9.48 ± 0.83	^a^ *
Hb [g/dL]	13.82 ± 0.50	14.32 ± 1.16	15.42 ± 0.54	19.69 ± 18.65	14.36 ± 0.60	21.32 ± 7.93	^-^
Hct [%]	44.42 ± 2.57 ^ab^	47.48 ± 3.34	50.84 ± 2.48 ^ac^	61.88 ± 48.22	46.30 ± 2.16 ^c^	50.10 ± 5.30 ^b^	^a^ *** ^bc^ *
MCV [fL]	48.61 ± 1.84 ^ab^	51.00 ± 1.38	51.89 ± 0.91 ^a^	50.93 ± 1.02	50.65 ± 2.22	52.86 ± 2.04 ^b^	^ab^ **
MCH [pg/cell]	15.19 ± 0.79	15.58 ± 0.48	15.58 ± 0.43	15.45 ± 0.37	15.75 ± 0.87	21.71 ± 7.54	
MCHC [g/dL]	31.19 ± 1.17	30.54 ± 0.40	30.05 ± 0.45 ^a^	30.39 ± 0.60	31.02 ± 0.68 ^a^	41.28 ± 14.69	^a^ *
WBC [10^3^/μL]	20.85 ± 8.80 ^abcd^	9.02 ± 3.54 ^a^	8.73 ± 1.32 ^a^	9.23 ± 1.05 ^c^	11.07 ± 2.85	9.09 ± 1.53 ^d^	^a b^ *** ^c^ * ^d^ **
PLT [10^3^/μL]	946.3 ± 202.8 ^ab^	637.2 ± 260.8	582.9 ± 287.3 ^a^	733.1 ± 253.3	889.4 ± 125.4 ^c^	506.9 ± 218.5 ^b c^	^a^* ^b^ *** ^c^ **
*n* = 12	**Biochemical Parameters**						
Glucose [mmoL/L]	11.51 ± 2.40	11.77 ± 1.03	11.99 ± 1.10	11.54 ± 1.80	11.80 ± 1.95	11.07 ± 2.18	-
Uric acid [mg/dL]	22.83 ± 11.29	21.00 ± 6.30	25.75 ± 6.61	23.58 ± 8.63	20.83 ± 5.84	22.75 ± 5.59	-
Urea [mmol/L]	8.48 ± 1.35	9.79 ± 2.38	11.81 ± 2.88	11.19 ± 4.00	10.17 ± 2.13	12.43 ± 4.32	-
Creatinine [μmol/L]	21.50 ± 4.98 ^ac^	20.92 ± 3.23	17.75 ± 1.96 ^ab^	17.08 ± 1.68 ^c^	23.44 ± 4.82 ^b^	20.10 ± 1.20	^abc^ *
ASPAT [U/L]	103.2 ± 28.7 ^abc^	87.2 ± 36.7	87.0 ± 19.8	69.6 ± 15.4 ^a^	72.6 ± 16.6 ^b^	71.9 ± 12.3 ^c^	^abc^ *
ALAT [U/L]	28.88 ± 11.44 ^abcd^	81.14 ± 36.96 ^a^	85.86 ± 27.57 ^be^	87.18 ± 62.90 ^c^	46.74 ± 18.85 ^e^	83.34 ± 32.11 ^d^	^e^ * ^ac^ ** ^bd^ ***
TG [mmoL/L]	0.79 ± 0.14 ^a^	0.83 ± 0.29 ^b^	0.48 ± 0.15 ^abc^	0.77 ± 0.31 ^c^	0.63 ± 0.13	0.66 ± 0.09	^c^ * ^b^ ** ^a^ ***
TC [mmoL/L]	2.51 ± 0.53 ^a^	2.26 ± 0.19	1.95 ± 0.25 ^abcde^	2.36 ± 0.16 ^b^	2.26 ± 0.59 ^cd^	2.31 ± 0.45 ^e^	^bcde^ * ^a^ ***
HDL [mmoL/L]	0.89 ± 0.07	0.96 ± 0.16	1.05 ± 0.78	1.01 ± 0.63	0.95 ± 0.50	0.81 ± 0.20	
PAL [U/L]	104.9 ± 48.7 ^abc^	147.1 ± 23.4	165.3 ± 26.1 ^b^	167.5 ± 23.2 ^a^	127.3 ± 35.0 ^d^	173.4 ± 16.0 ^cd^	^abd^ * ^c^ **

Mean values with the same superscript are significantly different between the indicated group at * *p* < 0.05; ** *p* < 0.01; *** *p* < 0.001.

**Table 5:** Correlation weights for the pairs of parameters based on PLS model (only the first fifteen correlation weights with highest absolute values were shown), see Section 2. for the explanation of all abbreviations.

Pairs of Correlated Parameters		Correlation Weights
MCV	MCH	0.200
MCV	follicular epithelial area	0.159
TEMP	fT3	0.155
ASPAT	fT3	0.150
MCV	overall thyroid area	0.149
RBC	uric acid	0.148
RBC	follicular luminal area	0.144
MCV	ASPAT	−0.143
fT3	follicular epithelial area	−0.147
IL-6	fT3	−0.147
fT3	overall thyroid area	−0.148
uric acid	Creatinine	−0.154
Creatinine	follicular luminal area	−0.157
RBC	Creatinine	−0.166
MCV	fT3	−0.188

**Table 6:** Influence of the breed of rats on the selected parameters in two in vivo models of hypothyroidism, caused by iodine deficiency (ID) or sulfadimethoxine (SDM) diet [4,15,17], see Section 2. for the explanation of the groups’ acronyms and all abbreviations.

Parameter		TSH	fT4	fT3	Temp	TC	TG	HDL	ASPAT	ALAT	GLU	WBC	PLT
**Wistar rats**	**ID**	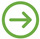	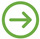	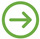	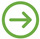	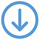	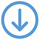	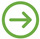	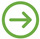	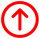	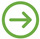	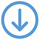	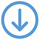
	**SDM**	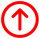	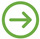	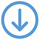	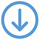	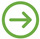	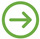	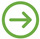	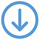	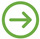	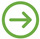	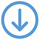	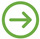
**Fischer rats**	**ID**	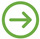	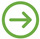	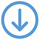	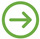	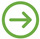	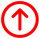	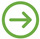	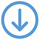	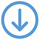	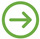	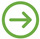	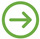
	**SDM**	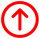	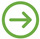	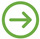	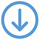	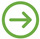	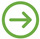	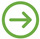	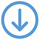	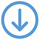	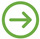	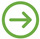	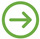

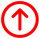
 increase 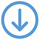 decrease 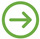 no changes.

**Table 7:** Influence of kohlrabi (KS), broccoli (BS) and rutabaga (RS) sprouts on the selected parameters in healthy rats of two different breeds [4,15,17], see Section 2. for the explanation of the groups’ acronyms and all abbreviations.

Parameter		TSH	fT4	fT3	Temp	TC	TG	HDL	ASPAT	ALAT	GLU	WBC	PLT
**Wistar rats**	**KS**	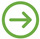	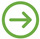	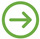	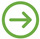	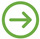	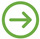	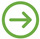	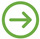	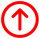	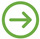	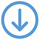	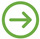
	**BS**	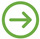	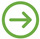	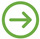	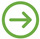	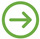	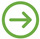	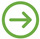	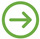	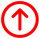	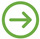	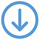	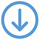
**Fischer rats**	**RS**	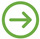	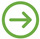	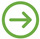	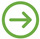	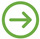	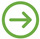	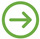	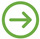	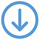	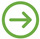	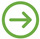	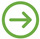

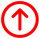
 increase 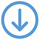 decrease 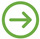 no changes.

## Data Availability

Data available upon request.
